# Large Language Models for Individualized Psychoeducational Tools for Psychosis: A Cross‐Sectional Study

**DOI:** 10.1111/eip.70230

**Published:** 2026-07-30

**Authors:** Musa Yilanli, Ian McKay, Daniel I. Jackson, Emre Sezgin

**Affiliations:** ^1^ Nationwide Children's Hospital Columbus Ohio USA; ^2^ The Ohio State University College of Medicine Columbus Ohio USA; ^3^ The Abigail Wexner Research Institute, Nationwide Children's Hospital Columbus Ohio USA

## Abstract

**Objective:**

This study aimed to evaluate the quality of GPT‐4–generated responses to commonly asked psychosis‐related psychoeducational questions from patients, caregivers and relatives in a first‐episode psychosis programme. Evaluation focused on accuracy, clarity, inclusivity, completeness, clinical utility and overall quality.

**Design:**

This cross‐sectional study employed a qualitative evaluation design. GPT‐4, accessed via the ChatGPT interface, generated responses to 20 psychosis‐related psychoeducational questions. These questions were developed through iterative discussion and consensus among clinicians working in a first‐episode psychosis treatment programme, informed by commonly encountered questions from patients, caregivers and relatives in clinical practice and are provided in Appendix A. The generated responses were subsequently evaluated for their potential clinical applicability.

**Primary Outcome:**

ChatGPT was presented with 20 psychoeducational questions derived from real‐world clinical interactions with patients, caregivers and relatives. Two experts in psychosis independently assessed the responses using a structured six‐domain rubric: accuracy (1–3), clarity (1–3), inclusivity (1–3), completeness (0–1), clinical utility (1–5) and overall quality (1–4), where lower scores indicate poorer performance and higher scores indicate stronger performance across domains. Discrepancies in ratings were resolved through discussion and consensus.

**Results:**

Using a structured evaluation rubric, LLM‐generated responses were assessed across accuracy, clarity, inclusivity, completeness, clinical utility and overall quality. Responses were generally coherent, well‐organized and readable across all 20 psychoeducational questions. Performance was strongest in accuracy (M ± SD = 2.88 ± 0.22), clarity (2.93 ± 0.18), completeness (0.93 ± 0.18) and clinical utility (4.35 ± 0.52), indicating that responses were largely correct, understandable and clinically relevant. Inclusivity scores were comparatively lower (2.30 ± 0.41). A descriptive linguistic analysis showed that responses were written at a relatively high Flesch–Kincaid Grade Level (FKGL) (mean = 15.59 ± 1.59), indicating increased reading complexity. Although responses addressed core aspects of the questions, some lacked sufficient nuance for complex or individualized clinical scenarios.

**Conclusions:**

GPT‐4, as an example of a large language model (LLM), may have a limited adjunctive role in supporting psychoeducation for psychosis when used within structured and clinician‐guided contexts. Although responses were generally readable and clinically relevant, their complexity and variability in inclusivity highlight potential limitations in accessibility for diverse patient populations, and cautious use is warranted given the ongoing concerns regarding accuracy, safety and real‐world implementation. Further research is needed before broader clinical integration can be recommended.

## 1 Introduction

Psychosis, characterized by a disruption in reality testing, presents a significant challenge for individuals and healthcare systems alike (Perrotta 2020). Effective education empowers patients to understand their condition, manage symptoms and navigate the complexities of treatment. However, traditional resources often fall short in accessibility, engagement and personalization to individual patient and caregiver needs (Spallek et al. 2023).

This study investigates the potential of GPT‐4 (via the ChatGPT interface), a large language model (LLM), to enhance patient education for psychosis. LLMs, trained on massive datasets of text and code, offer a promising avenue for developing interactive and adaptable learning tools. However, concerns regarding the accuracy and reliability of LLM‐generated information remain critical (Lundin et al. 2023). The quality of training data significantly influences LLM output, and the vast amount of information available online presents challenges in ensuring factual accuracy and adequate clinical nuance (Fakhoury 2019; Lee et al. 2021). These limitations are particularly important in psychiatric populations, where misinterpretation or overgeneralization of information may have clinically meaningful consequences. Although direct empirical evidence remains limited, emerging concerns suggest that AI‐generated responses may reinforce maladaptive or delusional beliefs in vulnerable individuals, given the established cognitive mechanisms underlying belief formation in psychosis and known safety considerations related to digital mental health tools (Freeman and Garety 2014; Torous et al. 2021).

To address these concerns, this study evaluates the performance of a specific LLM through expert assessment in the context of psychosis education. We analysed the LLM's responses to 20 psychoeducational questions developed through iterative discussion and consensus among clinicians in a first‐episode psychosis programme, reflecting commonly encountered real‐world questions from patients, caregivers and relatives. Our assessment focused on accuracy, clarity of communication, inclusivity of language, completeness of information, clinical utility and overall quality of the LLM‐generated responses. A structured scoring rubric was used, where lower scores indicate poorer performance and higher scores indicate stronger performance across domains.

This evaluation will provide valuable insights into the potential role and limitations of utilizing LLM‐based chatbots for psychosis education in clinical settings.

## 2 Methods

We used GPT‐4 (via the ChatGPT interface) as the LLM for this study. A set of 20 psychosis‐related psychoeducational questions was developed through iterative discussion and consensus among clinicians working in a first‐episode psychosis treatment programme. These questions were informed by commonly encountered inquiries from patients, caregivers and relatives in routine clinical care and are provided in full in Appendix A.

Each question was entered into ChatGPT in a new, independent session to generate responses. To assess response consistency, the same question was re‐prompted three times for the first three questions, and outputs were compared qualitatively for variability.

Responses were independently evaluated by two experts in psychosis (authors M. Y. and I. M.) using a structured six‐domain rubric. The evaluation domains included accuracy, clarity, inclusivity, completeness, clinical utility and overall quality. Discrepancies between raters were resolved through discussion and consensus.

Each domain was assessed using predefined scoring criteria. Accuracy, clarity and inclusivity were rated on 3‐point ordinal scales; completeness was assessed dichotomously (0–1) to indicate whether all key elements of the question were addressed; clinical utility was rated on a 5‐point Likert scale ranging from strongly disagree to strongly agree; and overall quality was rated on a 4‐point scale reflecting a global expert assessment. Higher scores indicated better performance across all domains. The overall quality score represented a holistic evaluation integrating all domains rather than a calculated composite score.

The rubric domains were informed by prior literature on health information quality, patient‐centred communication and evaluation of LLM‐generated content in healthcare contexts, including recent work examining the use of LLMs for caregiver information needs (Sezgin et al. 2025), as well as expert consensus. Formal validation of the rubric was beyond the scope of this study.

Descriptive statistics, including means and standard deviations, were calculated for each domain to summarize performance across the 20 questions.

In addition to expert evaluation, a descriptive linguistic analysis of GPT‐4‐generated responses was conducted. Metrics included readability (Flesch–Kincaid Grade Level [FKGL]), total character count, word count, number of unique words and number of sentences. These measures were used to provide additional context regarding the complexity, length and variability of the generated psychoeducational content.

**Table:** 

Rubric category	Question	Description	Grading scale
Accuracy	Is the response an accurate reply to the question?	Assesses whether the response directly and correctly addresses the question with accurate information.	1 = Inaccurate 2 = Partially accurate 3 = Accurate
Clarity	Is the message clearly conveyed?	Evaluates how easily understandable the response is, including structure, readability and avoidance of unnecessary jargon.	1 = No 2 = Partially 3 = Yes
Inclusivity	Is the response appropriate for a diverse range of recipients?	Assesses whether the response is culturally sensitive and applicable across diverse populations, including variation in cultural, racial and socioeconomic contexts.	1 = No 2 = Partially 3 = Yes
Completeness	Does the response fully answer the question?	Evaluates whether all key elements of the question are addressed.	0 = Incomplete 1 = Complete
Clinical utility	Is the response useful in a clinical context?	Assesses the practical usefulness of the response for patient and caregiver education in clinical settings.	1 = Strongly disagree 2 = Disagree 3 = Neutral 4 = Agree 5 = Strongly agree
Overall quality	What is the overall quality of the response?	Global expert assessment integrating accuracy, clarity, inclusivity, completeness and clinical utility.	1 = Very low 2 = Low 3 = Moderate 4 = High

*Note:* Higher scores indicate better performance across all domains. The overall quality score represents a global expert assessment and is not a calculated composite.

## 3 Results

LLM‐generated responses were generally coherent, organized and readable across the 20 psychoeducational questions. Across domains, responses demonstrated strong performance in accuracy (mean = 2.88, SD = 0.22) and clarity (mean = 2.93, SD = 0.18), indicating that responses were largely correct and easy to understand. Inclusivity scores were comparatively lower (mean = 2.30, SD = 0.41), suggesting variability in addressing diverse cultural and contextual perspectives.

Completeness was high (mean = 0.93, SD = 0.18), indicating that most responses addressed the key elements of the questions. Clinical utility was also rated favourably (mean = 4.35, SD = 0.52), reflecting that responses were generally perceived as useful in clinical contexts. The overall quality score was high (mean = 3.55, SD = 0.46), indicating generally favourable ratings across domains. Given that higher scores indicate better performance, these findings suggest stronger performance in accuracy, clarity, completeness and clinical utility, with relatively lower performance in inclusivity.

Median, interquartile range (IQR) and range values were consistent with mean findings, with scores generally clustered towards the higher end of each scale (Table 1).

**TABLE 1:** Summary of expert evaluation of GPT‐4–generated responses.

Metric	Accuracy	Clarity	Inclusivity	Completeness	Clinical utility	Overall quality
Mean (SD)	2.88 (0.22)	2.93 (0.18)	2.30 (0.41)	0.93 (0.18)	4.35 (0.52)	3.55 (0.46)
Median	3.00	3.00	2.00	1.00	4.50	3.50
IQR	0.4	0.0	0.5	0.0	0.9	0.9
Min–max	2.5–3.0	2.5–3.0	2.0–3.0	0.5–1.0	3.5–5.0	2.5–4.0
Scale range	1–3	1–3	1–3	0–1	1–5	1–4

*Note:* Higher scores indicate better performance across all domains. The overall quality score reflects a global expert assessment rather than a calculated composite.

Exploratory analysis using Spearman's rank correlation demonstrated a moderate positive association between clarity and completeness (ρ = 0.61, *p* = 0.004), as well as between clarity and clinical utility (ρ = 0.54, *p* = 0.013). These findings suggest that clearer responses were more likely to be perceived as complete and clinically useful; however, these associations should be interpreted cautiously given the exploratory nature of the analysis and the limited sample size.

A descriptive analysis of linguistic characteristics is presented in Table 2. On average, responses were written at an FKGL of 15.59 (SD = 1.59), indicating relatively high reading complexity. Responses contained a mean of 330 words (SD = 75.85) and 31.5 sentences (SD = 10.81), reflecting substantial length and detail.

**TABLE 2:** Linguistic and readability characteristics of GPT‐4–generated psychoeducational responses.

Question	FKGL	Characters	Words	Unique words	Sentences
1	15.8	2427	326	158	31
2	18.1	2244	323	168	21
3	12.5	1778	230	152	31
4	16.3	2656	338	166	32
5	13.7	2949	427	233	38
6	14.7	3523	481	218	46
7	14.8	2341	332	151	31
8	16.6	1590	225	128	18
9	17.1	2634	359	177	29
10	16.4	1404	189	113	16
11	16.2	2464	354	170	28
12	15.8	1971	272	160	26
13	18.8	1345	191	115	8
14	16.6	2454	326	166	27
15	13.6	2585	375	184	42
16	15.7	2769	380	198	33
17	14.1	2732	352	196	48
18	16.2	2765	399	198	35
19	13.5	2924	373	217	50
20	15.3	2587	348	192	40

Mean (SD)	15.59 (1.59)	2407.1 (551.21)	330 (75.85)	173 (32.67)	31.5 (10.81)
Median	15.8	2464	326	166	31
IQR	1.9	564.5	63.25	40	11.75
Min–max	12.5–18.8	1345–3523	189–481	113–233	8–50

Abbreviations: FKGL = Flesch–Kincaid Grade Level; IQR = interquartile range; SD = standard deviation.

Importantly, these findings suggest that while responses were comprehensive and informative, their reading level may exceed that appropriate for some patient and caregiver populations, potentially limiting accessibility in real‐world clinical use.

## 4 Discussion

This study examined how well GPT‐4 responds to common psychoeducational questions about psychosis and found generally strong performance across several domains. Overall, the responses were accurate, clearly written and addressed key aspects of the questions, suggesting that LLM‐generated content may offer a potentially accessible and understandable source of information for patients and caregivers. These findings align with growing evidence that LLMs can support patient‐facing communication, including recent work highlighting their usefulness for caregiver information needs (Sezgin et al. 2025). At the same time, research focused specifically on psychosis remains limited, highlighting the importance of evaluating these tools within this clinical context.

In contrast, inclusivity was an area where performance was less consistent, with responses varying in how well they accounted for cultural, social and contextual differences. Although many responses offered broadly applicable information, some did not fully capture the diversity of patient experiences or the contextual factors that shape psychosis. This is important in clinical practice, as sociocultural influences can affect how symptoms are understood, experienced and addressed. The linguistic analysis further supports these findings. While responses were generally detailed and comprehensive, they were written at a relatively high reading level, which may limit accessibility for individuals with lower health literacy. This highlights an additional challenge in applying LLM‐generated psychoeducation in clinical settings, where readability and comprehension are essential for effective patient engagement. Taken together, these findings suggest that while LLM‐generated responses may be helpful at a general level, their usefulness may be more limited in individualized or context‐specific situations.

These findings should also be considered in the light of broader clinical limitations. Although responses were generally accurate and clearly communicated, they may not provide the level of nuance needed for more complex or high‐risk scenarios and cannot replace individualized clinical assessment. In addition, there are ongoing concerns about how individuals with psychosis may interpret or engage with AI‐generated content. Given the known cognitive vulnerabilities in psychosis, there is a risk that certain responses could be misinterpreted or inadvertently reinforce maladaptive beliefs (Freeman and Garety 2014; Torous et al. 2021). This highlights the need to balance the accessibility of these tools with careful attention to safety.

Recent literature further underscores these concerns. Reports of AI‐induced psychotic experiences suggest that generative AI systems may, in some cases, reinforce or elaborate delusional beliefs (Østergaard 2023, 2025). This phenomenon has been linked to the tendency of LLMs to produce responses that align with user input, sometimes described as ‘sycophantic’ behaviour. In the context of psychosis, this represents a meaningful clinical risk and points to the importance of cautious implementation, appropriate safeguards and clinician involvement.

Several limitations should be considered when interpreting these findings. The study was based on a relatively small set of 20 questions from a single clinical setting, which may limit generalizability. The use of expert ratings introduces some subjectivity, and formal inter‐rater reliability was not assessed. In addition, the cross‐sectional and exploratory design does not allow conclusions about real‐world use, patient outcomes or safety.

Future research should move beyond expert evaluation to explore how patients and caregivers actually use and interpret LLM‐generated information. This includes examining outcomes such as comprehension, trust and potential behavioural impact, as well as how these tools perform in real‐world clinical settings. Further work is also needed to improve inclusivity and to develop safeguards for sensitive or delusion‐related content, particularly for use in higher‐risk populations.

From a clinical perspective, these findings suggest that LLMs may have a role as a supplementary source of psychoeducation. However, their use should remain within clinician‐guided contexts until more evidence is available regarding safety, effectiveness and appropriate implementation.

## 5 Conclusion

LLMs such as GPT‐4 may have a limited adjunctive role in supporting psychoeducation for psychosis, particularly in improving access to clear and understandable psychoeducational information for patients and caregivers. In this study, responses demonstrated strong performance in accuracy, clarity, completeness and clinical utility, with comparatively lower performance in inclusivity.

However, these findings should be interpreted cautiously. LLMs are not a substitute for clinical care, and important concerns remain regarding variability in response quality, limited nuance in complex clinical scenarios and potential unintended effects in vulnerable populations.

Further research is needed to evaluate safety, effectiveness and real‐world implementation before broader clinical integration can be recommended.

## Funding

The authors have nothing to report.

## Data Availability

The data that support the findings of this study are available from the corresponding author upon reasonable request.

## Appendix A Psychoeducational Questions

The following 20 psychoeducational questions were developed through iterative discussion between psychiatrists and psychologists working in a primary first‐episode psychosis treatment centre. The questions reflect commonly asked patient and caregiver questions encountered in this clinical setting.

1. Are there differences in long‐term outcomes between individuals with psychotic experiences and individuals with a psychotic disorder?
2. What methods are frequently used to diagnose psychotic disorders?
3. What are early warning signs of psychosis?
4. Is there added benefit from participating in a specialized psychosis clinic compared with general outpatient treatment?
5. How can I support a loved one with psychosis?
6. Are there considerations when deciding between medications, psychotherapy or a combination of both?
7. Are people with psychosis dangerous?
8. Is psychosis genetic?
9. What environmental factors can contribute to the development of psychosis in vulnerable populations?
10. Can psychosis be cured?
11. When might hospitalization be necessary due to psychosis?
12. Can children develop psychosis?
13. Can THC use cause psychosis?
14. Is there any connection between nicotine use and psychosis?
15. What can families do to address the stigma associated with psychosis?
16. What causes schizophrenia?
17. What are the short‐term and long‐term adverse effects of antipsychotics?
18. What is the role of long‐acting injectable antipsychotics in the treatment of psychosis?
19. Outside of medications, what types of treatments are available for psychosis?
20. If someone does not respond to prescribed medications, what other options are available for psychosis?
